# Exposure to Type 1 and Type 2 Maternal Diabetes is Associated with Stage 3-5 Retinopathy of Prematurity

**DOI:** 10.1016/j.xops.2026.101143

**Published:** 2026-03-04

**Authors:** Adam Lewis, Isaac Bakis, Alexa Love, Jennifer Sucre, Sara Lynn Van Driest, Lisa Bastarache, Dolly A. Padovani-Claudio

**Affiliations:** 1 Department of Biomedical Informatics, Vanderbilt University Medical Center, Nashville, Tennessee; 2 Vanderbilt University School of Medicine, Nashville, Tennessee; 3 Department of Pediatrics, Vanderbilt University Medical Center, Nashville, Tennessee; 4 *All of Us* Research Program, National Institutes of Health, (All Work Completed While at Vanderbilt University Medical Center), Bethesda, Maryland; 5 Department of Ophthalmology, University of Michigan, Ann Arbor, Michigan

**Keywords:** Retinopathy of prematurity, Maternal diabetes, Necrotizing enterocolitis, Bronchopulmonary dysplasia, Intraventricular hemorrhage

## Abstract

**Purpose:**

Pathologic similarities between retinopathy of prematurity (ROP) and diabetic retinopathy, leading causes of blindness, suggest that in utero exposure to maternal hyperglycemia may influence an infant’s risk of ROP progression to vision-threatening stages. Prior studies exploring this association yielded conflicting results and did not explore maternal diabetes mellitus (DM) subtypes. We aim to clarify these associations while adjusting for significant comorbidities.

**Design:**

Retrospective cohort study.

**Participants:**

Preterm infants born at <31 weeks gestational age (GA) or <1500 g birth weight (BW) receiving care at Vanderbilt University Medical Center’s Neonatal Intensive Care Unit between 2004 and 2021 with documented ROP staging and maternal diabetes status.

**Methods:**

Review of data from the Vanderbilt Neonatal Clinical Repository supplemented by chart review. Data analyzed by descriptive statistics of prevalence, BW, and GA for the cohorts as well as through multivariate logistic regression analyses with ROP stage and maternal diabetes as dependent and independent variables while adjusting for GA, BW, sex, race, year of birth, birth center location, necrotizing enterocolitis, intraventricular hemorrhage, and bronchopulmonary dysplasia. Infants with ROP stage 3-5 were considered cases, and those with ROP stage 0 were used as controls.

**Main Outcome Measures:**

The association between exposure to maternal diabetes and the presence of stage 3-5 versus stage 0 ROP.

**Results:**

Two thousand one hundred twenty-one (68%) infants had stage 0 ROP, and 311 (10%) had stage 3-5 ROP. Maternal DM prevalence was 9% and resulted in higher BW and GA in premature infants. Lower BW and GA were associated with more severe ROP. Stage 3-5 ROP was significantly associated with maternal DM (odds ratio [OR] 3.04, 95% confidence interval [CI]: 1.67–5.45, *P* = 0.000218), including type 1 DM (T1DM) (OR 6.36, 95% CI 1.29–28.29, *P* = 0.0174) and type 2 DM (T2DM) (OR 5.82, 95% CI 2.06–15.71, *P* = 0.00066).

**Conclusions:**

Our results suggest that maternal diabetes, including T1DM and T2DM, increase an infant’s risk of developing vision-threatening ROP. The BW and GA profiles of infants with maternal diabetes may provide false reassurance regarding their risk of progression. Considering maternal DM exposure in screening decisions (inclusion and intervals) may help to preserve vision in at-risk infants.

**Financial Disclosure(s):**

Proprietary or commercial disclosure may be found in the Footnotes and Disclosures at the end of this article.

Retinal vascularization is not complete until approximately 36–40 weeks of gestation; thus, premature infants have incompletely vascularized retinas at birth.^1^ As their retina develops ex utero, the distribution, organization, and integrity of the expanding retinal vasculature can be disrupted, leading to retinopathy of prematurity (ROP), a leading cause of childhood blindness.^2^ Preretinal neovascularization, which is driven by ischemia in the remaining avascular retina from disrupted normal vascular growth in ROP, also manifests in patients with diabetes where the ischemia is in areas of capillary dropout.^3^ In both conditions, screening is important to detect advanced stages with preretinal neovascularization requiring treatment (i.e., anti-VEGF therapy and laser) to reduce the likelihood of severe visual impairment.^3^, ^4^, ^5^, ^6^, ^7^

Given that ROP and proliferative diabetic retinopathy both present with preretinal neovascularization, that pregnancy exacerbates retinopathy progression in women with diabetes,^8^ and that circulating blood factors, including glucose, freely cross the placenta,^9^ we suspect that maternal diabetes exposure would predispose the developing retina to retinopathy when a child is born prematurely. The American Academy of Pediatrics and American Academy of Ophthalmology have outlined screening guidelines for all infants with a birth weight (BW) ≤1500 g or a gestational age (GA) ≤30 weeks, as historically low BW and GA are strongly associated with ROP progression.^10^ Several comorbid infant conditions, including necrotizing enterocolitis (NEC), bronchopulmonary dysplasia (BPD), and intraventricular hemorrhage (IVH) are also associated with increased risk of ROP progression.

Infants outside BW and GA guidelines can be screened for ROP if the care team considers them high risk.^11^ Although Tunay et al^12^ found increased risk of ROP for infants with BW > 1500 g who were exposed to maternal diabetes, maternal diabetes exposure is not typically a consideration. Furthermore, the few studies exploring this association for children <1500 g BW have yielded inconsistent results, and none have explored the association with the different subtypes of maternal diabetes.^13^, ^14^, ^15^, ^16^

We abstracted a cohort of 3139 dyads of mothers and infants <1500 g BW and <31 weeks GA screened for ROP from a large, curated database of >20 000 infants to test for an association between maternal diabetes and infant diagnosis of vision-threatening ROP (stage 3-5) versus no detectable retinopathy (stage 0). We further explored whether specific diabetes subtypes (gestational DM [GDM], type 1 DM [T1DM], or type 2 DM [T2DM]) contribute to risk of ROP advancement to stages 3-5. Clarifying the contribution of hyperglycemia exposure to ROP risk may inform refinement of ROP monitoring and management decisions to decrease the risk of visual morbidity in premature infants.

## Methods

### Study Design

**Figure 1:**
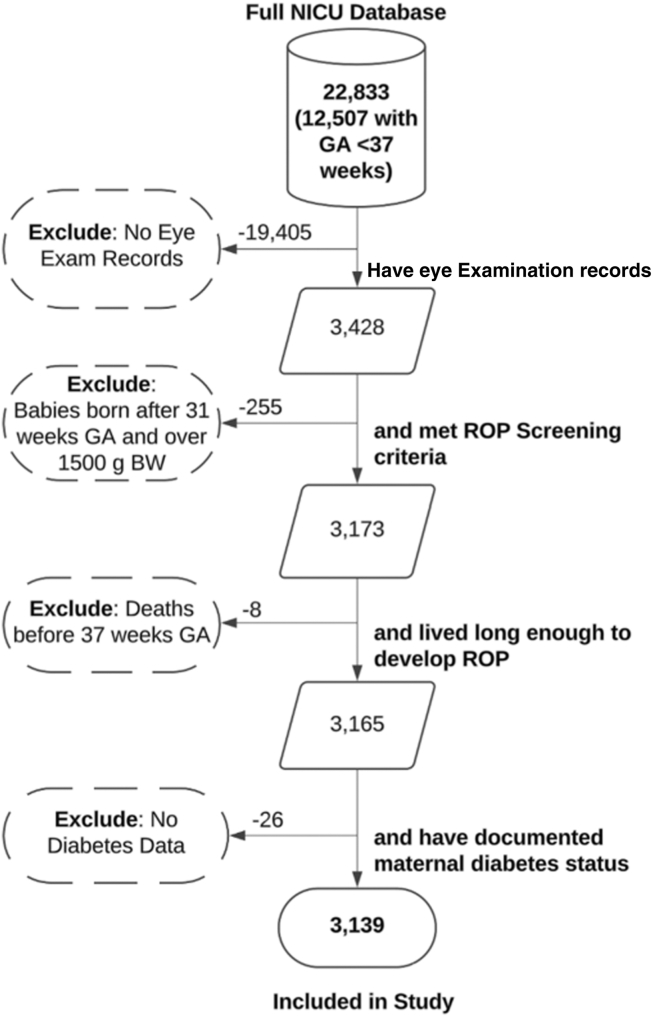
Inclusion/exclusion workflow. BW = birth weight; GA = gestational age; NICU = Neonatal Intensive Care Unit; ROP = retinopathy of prematurity.

This is a retrospective cohort study of preterm infants receiving care at Vanderbilt University Medical Center’s Neonatal Intensive Care Unit (NICU) aimed at exploring the association between maternal DM presence and type and diagnosis of stage 3-5 ROP versus stage 0 ROP. The study included infants of ≤ 30 weeks and 6 days GA at birth (<31 weeks GA) or with BW ≤1500 g who survived at least 30 days of life or 40 weeks corrected GA. We further required evidence of a ROP screening exam and ascertainment of maternal diabetes data (Fig 1). Data access was reviewed and approved by Vanderbilt University Medical Center’s Institutional Review Board (IRB#201022 and #201843), Nashville, Tennessee. Given the retrospective nature, no informed consent was required. The study adhered to the principles outlined in the Declaration of Helsinki.

### Study Population and Data Sources

We utilized clinical data from the Vanderbilt NICU Neonatal Clinical Repository.^17^ Vanderbilt maintains this database as part of the Vermont Oxford Network, a national quality improvement initiative that collects perinatal data for NICU patients that has been used in prior research projects.^17^^,^^18^ The prospectively ascertained database includes 270 clinical variables from 22833 infants admitted to the NICU at Vanderbilt. These data have been collected and curated by trained research coordinators. Retinopathy of prematurity variables in the database include zone, stage, and treatment, but not plus disease status. For the purposes of this study, we pulled data from 2004–2021 for the following variables: highest ROP stage, maternal diabetes presence and type (GDM, T1DM, T2DM), infant GA at birth, BW, sex, electronic health records (EHRs) race, year of birth, presence of NEC, IVH, and BPD, and whether the infant was born at our institution (defined as inborn) or outside our institution (defined as outborn). We also manually screened all included patients who were not diseased for the presence of plus disease.

### Cohort Definition

Retinopathy of prematurity is usually defined by stage, zone, and vascular characteristics after dilated fundus exam is performed by a trained ophthalmologist.^19^ Retinopathy of prematurity stage, our primary outcome variable, is defined by the vertical distribution of the nascent retinal vessels (within or outside of the retina) and by the attachment state of the retina. Stage 3 denotes preretinal neovascularization, and stages 4 and 5 indicate partial and total retinal detachment. Retinopathy of prematurity stage 0 represents no detectable retinopathy. Retinopathy of prematurity staging was made according to the International Classification of Retinopathy of Prematurity^7^ standards. In cases where the 2 eyes provided different stages, the highest stage of the worse eye was used. Retinopathy of prematurity stage is recorded in the database with both an ROP stage variable and with International Classification of Diseases 9 and 10 stage-specific codes based on the ROP diagnosis provided by an ophthalmologist after dilated fundus examination. The ROP stage variable was manually verified against International Classification of Diseases codes. Any discrepancies were resolved by manual chart review by our team.

Given that ROP is a progressive disease with a degree of overlap, we defined the Boolean variable “severe ROP” to include as cases all infants with ROP stage 3 through 5 (considered severe or vision-threatening since these stages most often require treatment) and defined as controls those with stage 0 (undetectable disease). BW, GA, and Z-score data are presented for all stages, and stages 1 and 2 were included in the subanalysis using ROP as a continuous variable. All available EHR’s of surviving infants deemed to have ROP stage 3-5, as well as all of those with documented exposure to maternal diabetes, were further manually reviewed to confirm ROP staging and treatment. Records were also reviewed when comorbidity data were missing. By default, unless irrefutable new staging evidence was found during chart review, values extracted from the database were used to define the cohorts.

We calculated Fenton Z-scores for all but 7 patients, which had GA < 22 weeks and lie below the lowest limit of the growth chart. These patients were excluded from median Fenton z-score percentile data only.

### Statistical Analysis

We summarized the following variables: race, sex, inborn/outborn status, maternal DM, ROP stage, NEC, IVH, and BPD, with counts and percentages, stratifying the data by either ROP stage or by maternal diabetes status. We also summarized the median and interquartile ranges of estimated GA and BW for each variable. We tested significant differences between prevalence of cases and controls for each variable subcategory using chi-squared test (or Fisher exact test if there were values less than 5). Differences in median BW and GA between infants with ROP stage 0 vs. 3-5, and between infants born to mothers with or without diabetes were assessed using Wilcoxon signed-rank test. Differences in median BW and GA in each subcategory were analyzed using Kruskal–Wallis which compares the distributions of a continuous variable across independent groups. Finally, we used GraphPad Prism to plot trends in the relationship between GA and BW for various ROP stages, treatment, demographics, and comorbidities (including maternal diabetes exposure) with their Fenton z-score^20^ median percentiles per category.

We then used multivariate logistic regression analyses to assess the relationship between maternal diabetes exposure and ROP stage severity. On our first analysis, ROP status was the dependent variable and maternal diabetes the independent variable. A follow-up analysis assessed comorbid associations with maternal diabetes using logistic regression with maternal diabetes as the dependent variable, and ROP as an independent variable.

For these regression analyses, maternal DM exposure was analyzed either as a Boolean (yes/no) variable, or by subtype (T1DM, T2DM, GDM). Likewise, ROP severity was analyzed as a Boolean variable indicating stage 0 versus stage 3-5, or as a continuous variable. Neonates with ROP stages 1 and 2 were excluded from analyses, where ROP was defined as a Boolean variable. In both regression models, we included covariates for GA, BW, sex, age, race, NEC, IVH, BPD, birth year as a continuous variable, and birth center location (inborn versus outborn).

Because care practices have changed over the duration of our study and heterogeneity is possible across hospitals, we conducted 2 sensitivity analyses. First, we conducted a stratified analysis of inborn/outborn patients using the same multivariate regression model as described above for each subgroup. Second, we conducted the same regression analysis for babies born after 01/01/2012. All statistical analyses were performed using R (v.4.4.0), in which the lower limit for *P* values is 2.2 x 10^-16^.

## Results

### Demographic and Comorbid Patterns

Of the 22833 infants in the database, roughly half were premature (<37 weeks GA) and 3139 infants met the inclusion criteria for our study (Fig 1). As seen in Table 1, which illustrates counts for all demographic and clinical variables broken down by ROP stage, 311 infants (10%) had stage 3, 4, or 5 (our outcome of interest, which is considered vision-threatening). These were classified as cases. On the other hand, 2121 (69%) had stage 0 ROP, and were classified as controls. The fraction of infants of EHR reported White race was higher (77% vs. 71%) and of Black race lower (21% vs. 26%) in cases than in controls (X^2^ = 4.6982, *P* value = 0.03019). The proportion of females (46%–47%, X^2^ = 0.21289, *P* value = 0.6445) and of exposure to maternal diabetes (9%, X^2^ = 0.00054783, *P* value = 0.9813) was similar among cases and controls. Likewise, the distribution of maternal diabetes subtypes was not significantly different between the 193 infants from mothers with diabetes who were controls (stage 0 ROP, 60% GDM, 13% T1DM, and 27% T2DM), and the 29 that were cases (stage 3-5 ROP, 52% GDM, 14% T1DM, and 34% T2DM; Fisher exact *P* value = 0.817).

**Table 1:** Demographic and Comorbidity Prevalence by ROP Stage

Counts by ROP Stage																	
Variable	ROP St0		ROP St3-5		Chi-Square or ∗Fisher Exact Test *P* Value	ROP St1		ROP St2		ROP St3		ROP St4		ROP St5		All	
Total	2121		311			373		334		274		26		11		3139	
Race ∗	N	%	n	%	*0.089*	N	%	n	%	n	%	n	%	n	%	n	%
White	1496	71%	241	77%		278	75%	250	75%	213	78%	19	73%	9	82%	2265	72%
Black	554	26%	64	21%		89	24%	68	20%	55	20%	7	27%	2	18%	775	25%
Asian	29	1%	5	2%		3	1%	5	1%	5	2%	0	0%	0	0%	42	1%
Native Hawaiian	2	0%	0	0%		0	0%	1	0%	0	0%	0	0%	0	0%	3	0%
Other	35	2%	1	0%		2	1%	7	2%	1	0%	0	0%	0	0%	45	1%
Unknown	5	0%	0	0%		1	0%	3	1%	0	0%	0	0%	0	0%	9	0%
Sex	N	%	n	%	*0.645*	N	%	n	%	n	%	n	%	n	%	n	%
M	1119	53%	169	54%		182	49%	172	51%	150	55%	12	46%	7	64%	1642	52%
F	1002	47%	142	46%		191	51%	162	49%	124	45%	14	54%	4	36%	1497	48%
Birth location	n	%	n	%	*<2.2 x 10*^*-16*^	N	%	n	%	n	%	n	%	n	%	n	%
Inborn	1455	69%	88	28%		235	63%	182	54%	84	31%	4	15%	0	0%	1960	62%
Outborn	666	31%	223	72%		138	37%	152	46%	190	69%	22	85%	11	100%	1179	38%
Maternal DM	n	%	n	%	*0.981*	N	%	n	%	n	%	n	%	n	%	N	%
No maternal DM	1928	91%	282	91%		351	94%	307	92%	248	91%	24	92%	10	91%	2868	91%
Maternal DM	193	9%	29	9%		22	6%	27	8%	26	9%	2	8%	1	9%	271	9%
DM type ∗	n	%	n	%	*0.817*	N	%	n	%	n	%	n	%	n	%	N	%
GDM	115	5%	15	5%		11	3%	18	5%	13	5%	2	8%	0	0%	159	5%
T1DM	25	1%	4	1%		4	1%	6	2%	4	1%	0	0%	0	0%	39	1%
T2DM	53	2%	10	3%		7	2%	3	1%	9	3%	0	0%	1	9%	73	2%
NEC	n	%	n	%	*9.33 x 10*^*-12*^	N	%	n	%	n	%	n	%	n	%	N	%
No NEC	2009	95%	262	84%		337	90%	290	87%	232	85%	19	73%	11	100%	2898	92%
NEC	112	5%	49	16%		36	10%	44	13%	42	15%	7	27%	0	0%	241	8%
IVH	n	%	n	%	*<2.2 x 10*^*-16*^	N	%	n	%	n	%	n	%	n	%	N	%
No IVH	1536	72%	148	48%		242	65%	187	56%	130	47%	12	46%	6	55%	2113	67%
IVH grade 1	359	17%	45	14%		49	13%	54	16%	40	15%	4	15%	1	9%	507	16%
IVH grade 2	120	6%	36	12%		33	9%	46	14%	30	11%	6	23%	0	0%	235	7%
IVH grade 3	53	2%	32	10%		20	5%	17	5%	30	11%	0	0%	2	18%	122	4%
IVH grade 4	53	2%	50	16%		29	8%	30	9%	44	16%	4	15%	2	18%	162	5%
BPD	n	%	n	%	*<2.2 x 10*^*-16*^	N	%	n	%	n	%	n	%	n	%	N	%
No BPD	1026	48%	12	4%		71	19%	32	10%	10	4%	1	4%	1	9%	1141	36%
BPD	1095	52%	299	96%		302	81%	302	90%	264	96%	25	96%	10	91%	1998	64%

BPD = bronchopulmonary dysplasia; DM = diabetes mellitus; GDM = gestational diabetes mellitus; IVH = intraventricular hemorrhage; NEC = necrotizing enterocolitis; ROP = retinopathy of prematurity; T1DM = type 1 diabetes mellitus; T2DM = type 2 diabetes mellitus.

The table reports the number and percentage of infants within each variable including demographics (race, sex, birth location), exposure (maternal diabetes: type 1, type 2, gestational, none), and comorbidities (NEC, IVH, and BPD). Columns are stratified by ROP stage. *P* values are signified using italics, and variables with *P* values <0.05 are signified with an asterisk (∗).

Most of the infants in the stage 3-5 group were born outside of Vanderbilt (72%). In contrast, most controls (69%) were inborn (*P* value < 2.2e–16). Comorbidities were higher in the cases that had controls; this included NEC (15% vs. 5%, *P* value = 9.332e–12), BPD (90% vs. 51%, *P* value < 2.2e–16), and IVH grade 4 (16% vs. 2% *P* value < 2.2e–16). Interestingly, children with exposure to maternal diabetes had lower rates of these comorbidities than those from mothers without diabetes (i.e., NEC [6.6% vs. 7.7%, *P* value = 0.582], BPD [60% vs. 64%, *P* value = 0.2347 ], and IVH grade 4 [1.5% vs. 5.5% *P* value = 0.03154]) and were more likely to be inborn (70% vs. 62%, *P* value = 0.005215 Table 2), although only the differences in BPD, IVH, and birth location were statistically significant.

**Table 2:** Demographic, ROP Stage, and Comorbidity Prevalence by Maternal Diabetes Exposure Status

Counts by Maternal Diabetes Exposure													
Variable	No Diabetes		Yes Diabetes		Chi-Square or ∗Fisher Exact Test *P* Value	GDM		T1D		T2D		All	
Total	2868		271			159		39		73		3139	
Race ∗	n	%	N	%	*0.517*	n	%	n	%	n	%	N	%
White	2073	72%	192	71%		115	72%	31	79%	46	63%	2265	72%
Black	705	25%	70	26%		38	24%	8	21%	24	33%	775	25%
Asian	40	1%	2	1%		2	1%	0	0%	0	0%	42	1%
Native Hawaiian	3	0%	0	0%		0	0%	0	0%	0	0%	3	0%
Other	38	1%	7	3%		4	3%	0	0%	3	4%	45	1%
Unknown	9	0%	0	0%		0	0%	0	0%	0	0%	9	0%
Sex	n	%	N	%	*0.873*	n	%	n	%	n	%	N	%
M	1502	52%	140	52%		87	55%	20	51%	33	45%	1642	52%
F	1366	48%	131	48%		72	45%	19	49%	40	55%	1497	48%
Birth location	n	%	N	%	*0.005*	n	%	n	%	n	%	N	%
Inborn	1769	62%	191	70%		110	69%	25	64%	56	77%	1960	62%
Outborn	1099	38%	80	30%		49	31%	14	36%	17	23%	1179	38%
ROP stage ∗	n	%	N	%	*0.395*	n	%	n	%	n	%	N	%
ROP stage 0	1928	67%	193	71%		115	72%	25	64%	53	73%	2121	68%
ROP stage 1	351	12%	22	8%		11	7%	4	10%	7	10%	373	12%
ROP stage 2	307	11%	27	10%		18	11%	6	15%	3	4%	334	11%
ROP stage 3	248	9%	26	10%		13	8%	4	10%	9	12%	274	9%
ROP stage 4	24	1%	2	1%		2	1%	0	0%	0	0%	26	1%
ROP stage 5	10	0%	1	0%		0	0%	0	0%	1	1%	11	0%
ROP category	n	%	N	%	*0.185*	n	%	n	%	n	%	N	%
0	1928	67%	193	71%		115	72%	25	64%	53	73%	2121	68%
1, 2	658	23%	49	18%		29	18%	10	26%	10	14%	707	23%
3, 4, 5	282	10%	29	11%		15	9%	4	10%	10	14%	311	10%
NEC	n	%	N	%	*0.582*	n	%	n	%	n	%	N	%
No NEC	2645	92%	253	93%		149	94%	36	92%	68	93%	2898	92%
NEC	223	8%	18	7%		10	6%	3	8%	5	7%	241	8%
IVH ∗	n	%	n	%	*0.016*	n	%	n	%	n	%	N	%
No IVH	1918	67%	195	72%		111	70%	29	74%	55	75%	2113	67%
IVH grade 1	460	16%	47	17%		28	18%	9	23%	10	14%	507	16%
IVH grade 2	217	8%	18	7%		11	7%	1	3%	6	8%	235	7%
IVH grade 3	115	4%	7	3%		5	3%	0	0%	2	3%	122	4%
IVH grade 4	158	6%	4	1%		4	3%	0	0%	0	0%	162	5%
BPD	n	%	n	%	*0.235*	n	%	n	%	n	%	N	%
No BPD	1033	36%	108	40%		59	37%	15	38%	34	47%	1141	36%
BPD	1835	64%	163	60%		100	63%	24	62%	39	53%	1998	64%

BPD = bronchopulmonary dysplasia; GDM = gestational diabetes mellitus; IVH = intraventricular hemorrhage; NEC = necrotizing enterocolitis; ROP = retinopathy of prematurity; T1D = type 1 diabetes; T2D = type 2 diabetes.

The table reports the number and percentage of infants within each variable including demographics (race, sex, birth location), outcome (ROP stages), and comorbidities (NEC, IVH, and BPD). Columns are stratified by exposure to maternal diabetes and subtypes. *P* values are signified using italics, and variables with *P* values <0.05 are signified with an asterisk (∗).

Table 2 illustrates counts for all demographic and clinical variables broken down by maternal diabetes exposure. Infants of mothers with T2DM had the highest proportion of inborn infants (chi-square test with three degrees of freedom comparing diabetes subtypes, X^2^ = 10.174, *P* value = 0.0172). Otherwise, there were no statistically significant differences in distributions.

Given that low birth weight is associated with ROP severity^21^ and that children from mothers with diabetes historically tend to be heavier than those without^22^, we looked at median BW data by ROP category (Supplemental Table 1, available at www.ophthalmologyscience.org) and by diabetes exposure (Supplemental Table 2, available at www.ophthalmologyscience.org). Further data corresponding to median GA stratified by ROP stage and by DM exposure can be found in Supplemental Tables 3 and 4, available at www.ophthalmologyscience.org, respectively.

Retinopathy of prematurity progression is known to be associated with decreased BW and GA.^21^ On average, infants with higher stages of ROP (as well as those with comorbidities such as IVH, BPD, and NEC) tended to have lower BW and GA, while infants exposed to maternal diabetes tended to have higher BW and GA than those without (Supplemental Tables 1–4).

**Figure 2:**
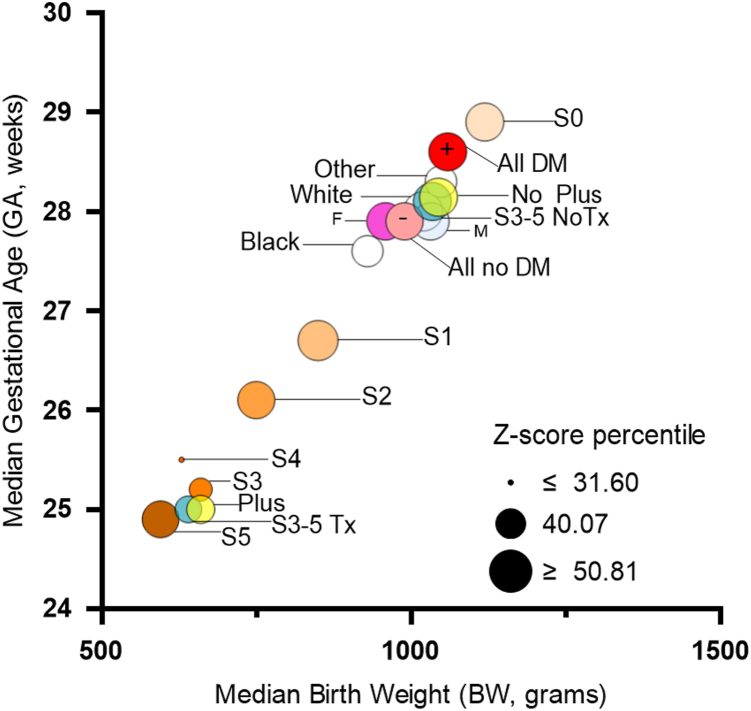
Median BW and GA distribution by demographics, maternal diabetes exposure, and ROP severity. Data are segregated using median BW and GA values for demographic groups (sex and age), maternal diabetes status (yes [+], no [–]), ROP staging [0, 1, 2, 3, 4, 5], plus disease, and for treatment (Tx) status (only for those with stage 3-5 ROP). Each circle is labeled with its corresponding group. Dot size indicates Fenton Z-score median percentile for that group. BW = birth weight; DM = diabetes mellitus; GA = gestational age; ROP = retinopathy of prematurity.

**Figure 3:**
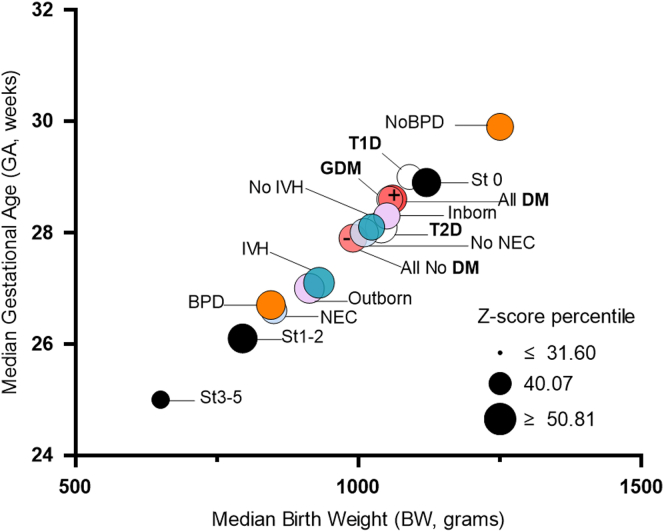
Median BW and GA distribution by ROP categories and comorbidities including maternal diabetes subtypes. Data are segregated using median BW and GA values for infants by maternal diabetes status (yes [+], no [–]) and subtypes (T1D, T2D, GDM), inborn/outborn status, presence or absence of comorbidities (BPD, NEC, IVH), and ROP plus disease status and staging (0, 1-2, 3-5) categories. D Dot size indicates Fenton Z-score median percentile for that group. BPD = bronchopulmonary dysplasia; BW = birth weight; DM = diabetes mellitus; GA = gestational age; GDM = gestational diabetes mellitus; IVH = intraventricular hemorrhage; NEC = necrotizing enterocolitis; ROP = retinopathy of prematurity; T1D = type 1 diabetes; T2D = type 2 diabetes.

To help visualize these and other trends, the GA and BW patterns for different demographic groups, ROP stages, and comorbidities including maternal DM and subtypes are plotted in Figures 2 and 3, where the size of each circle represents the median Fenton Z-score percentile for that category. Median (p50) with lower 25th (P25) and upper 25th (P75) quantiles of Z-score percentiles were 0.46 [0.22, 0.69] for all infants, 0.46 [0.24, 0.69] for infants with stage 0 ROP, and 0.33 [0.13–0.65] for infants with stage 3-5 ROP, suggesting ROP approaches small for GA Z-scores. Infants with Stage 3-5 ROP not only had lower GA and BW (both with *P* value< 2.2e–16), but also lower z-score percentiles than those with stage 0 (*P* value = 3.615e–05).

**Figure 4:**
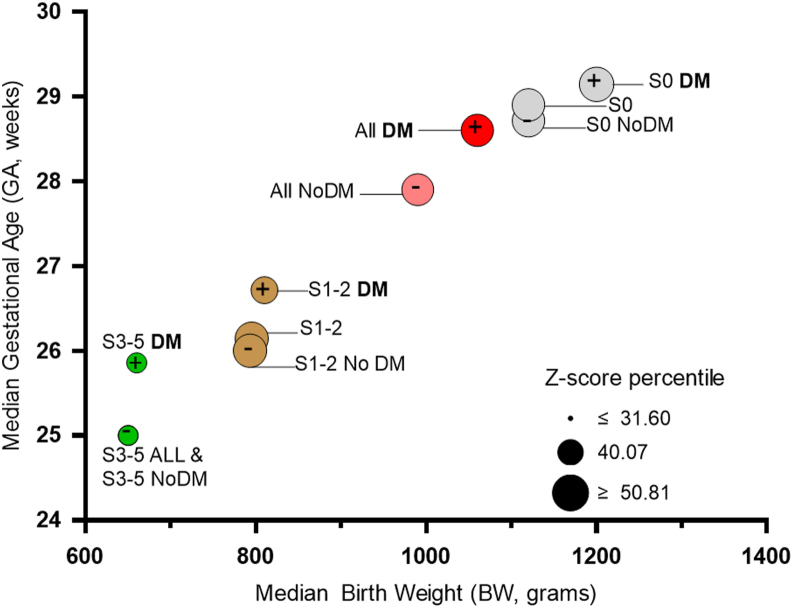
Median birth weight and GA of infants with different ROP stages segregated by maternal diabetes exposure. Birth weight and GA values for infants in each ROP stage category (S 0, 1-2, 3-5; plus or no plus) are subdivided by maternal diabetes status. Each circle is labeled with its corresponding group. Dot size indicates Fenton Z-score median percentile for that group. DM = diabetes mellitus; GA = gestational age; ROP = retinopathy of prematurity.

Finally, Figure 4 plots the distribution of BW and GA attributes for infants in each ROP stage category in relation to their maternal diabetes exposure status. The BW and GA in infants with exposure to maternal diabetes in each ROP category were higher than in those without, leaning towards profiles of children with less ROP progression. The higher GA in infants of mothers with diabetes was more pronounced for infants with more advanced stages of ROP, while the higher BW was more pronounced for controls with stage 0 ROP. Infants with ROP stage 3-5, especially those with maternal diabetes exposure, had the lowest z-scores (smallest circles in plots), consistent with association of poor outcomes with small for GA infants.

## Associations between ROP Severity and In Utero Exposure to Maternal Diabetes

**Figure 5:**
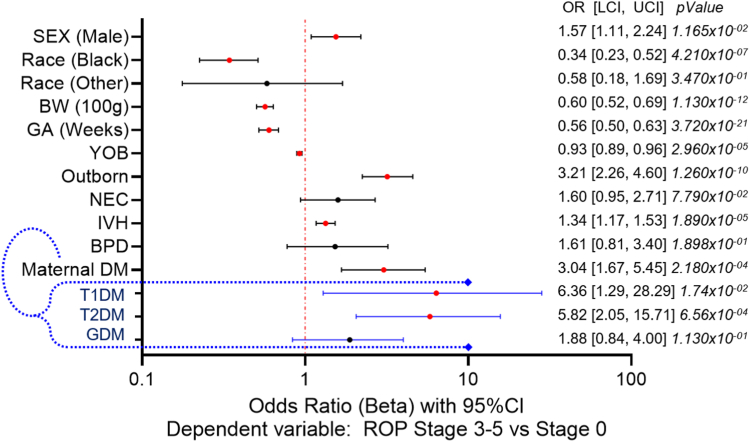
Multivariate logistic regression analysis for stage 3-5 ROP (vs. stage 0). Forest plot displaying the odds ratio and 95% confidence intervals from the multivariate regression analysis with severe ROP (stage 3-5 vs. stage 0) as the dependent variable and maternal diabetes as the independent variable. Reference values are sex (female), race (White), birth center location (inborn), NEC (no), IVH (stage 0), BPD (no), maternal diabetes (no). The results for the analysis including maternal diabetes subtypes are highlighted in blue. The vertical line represents no effect, odds ratios to the right of the line indicate a positive association, and odds ratios to the left indicate a protective association. Confidence intervals crossing the horizontal line indicate a failure to establish a significant association (black dots). *P* < 0.05 is statistically significant (red dots). BPD = bronchopulmonary dysplasia; BW = birth weight; CI = confidence interval; DM = diabetes mellitus; GA = gestational age; GDM = gestational diabetes mellitus; IVH = intraventricular hemorrhage; LCI = lower confidence interval; NEC = necrotizing enterocolitis; OR = odds ratio; ROP = retinopathy of prematurity; T1DM = type 1 diabetes mellitus; T2DM = type 2 diabetes mellitus; UCI = upper confidence interval; YOB = year of birth.

We used a multivariate logistic regression model to test for an association between prenatal exposure to maternal diabetes and ROP severity. A statistically significant positive association was found using exposure to maternal diabetes as the independent variable and the risk of stage 3-5 ROP as a dependent variable, controlling for potential confounding effects of sex, race, GA, BW, year of birth, birth center location, NEC, IVH, and BPD (odds ratio [OR]: 3.04 (95% confidence interval [CI]: 1.67, 5.45), *P* = 0.000218; Fig 5). Similar associations were found when maternal diabetes was stratified by subtype but were only statistically significant for T1DM (OR: 6.36 [95% CI: 1.29–28.29], *P* = 0.0174) and T2DM (OR: 5.82 [95% CI: 2.06–15.71], *P* = 0.00066). Low GA, low BW, earlier year of birth, male sex, being born outside our institution, and the presence of IVH were all also significantly associated with increased odds of stage 3-5 ROP in the multivariate model.

Although there was a trend toward association with GDM, it was not found to be statistically significant (OR: 1.88 [95% CI: 0.84–4.00], *P* = 0.113). However, the median GA of infants with advanced stages of ROP (25 weeks GA) is in the early range of the window for maternal GDM testing (24–28 weeks GA).

**Figure 6:**
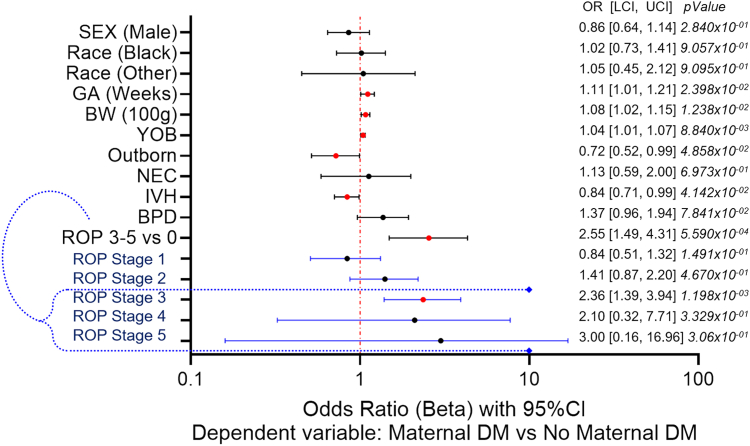
Multivariate logistic regression analysis results for associations with DM as the exposure. Forest plot displaying the odds ratio and 95% confidence intervals from the multivariate regression analysis with maternal diabetes exposure (yes vs. no) as the dependent variable and ROP as a dichotomous variable (stage 3-5 vs. stage 0) as the independent variable. Reference values are: sex (female), race (White), birth center location (inborn), NEC (no), IVH (stage 0), BPD (no), ROP stage (0). *P* < 0.05 is statistically significant. The results for the outcome of ROP as a continuous variable are highlighted in blue. The vertical line represents no effect, odds ratios to the right of the line indicate a positive association, and odds ratios to the left indicate a protective association. Confidence intervals crossing the horizontal line indicate a failure to establish a significant association (black dots). *P* < 0.05 is statistically significant (red dots). BPD = bronchopulmonary dysplasia; BW = birth weight; CI = confidence interval; DM = diabetes mellitus; GA = gestational age; IVH = intraventricular hemorrhage; LCI = lower confidence interval; NEC = necrotizing enterocolitis; OR = odds ratio; ROP = retinopathy of prematurity; UCI = upper confidence interval; YOB = year of birth.

When maternal diabetes was used as the independent variable and ROP as the dependent variable (Fig 6), a similar positive association was found. However, in this case, GA, BW, year of birth, inborn status, and the absence of IVH were positively associated with maternal diabetes exposure. This trend is opposite to ROP, where these variables had a negative association with the outcome.

Given that year and location of birth could be associated with differences in medical management, we performed sensitivity analyses for these variables (Supplemental Table 5, available at www.ophthalmologyscience.org). Our primary association was robust to inborn–outborn status as well as to our year of birth stratification. Maternal diabetes exposure continued to display a positive significant correlation with ROP in outborn (OR = 2.445794, *P* value = 0.029569) and inborn (OR = 3.667022, *P* value = 0.005) patients. Patients born after 2012 also replicated the positive significant association between maternal diabetes exposure and stage 3-5 ROP (OR = 4.33196, *P* value = 0.000146).

## Discussion

Multiple studies have attempted to determine additional ROP risk factors that might enhance the sensitivity and specificity of screening protocols to reduce ROP-associated visual morbidity.^10^^,^^11^^,^^23^, ^24^, ^25^, ^26^, ^27^, ^28^, ^29^ Our results add to the few reports exploring the association between maternal diabetes and the development of ROP, which have previously yielded conflicting results.^13^, ^14^, ^15^ Two studies showed positive correlations between maternal diabetes and severe ROP but did not look at maternal diabetes subtypes. One of these included infants with BW < 1500 g and the other included infants with BW > 1500 g.^12^^,^^13^ Two other studies, which also pooled all cases irrespective of DM type, did not detect significant associations between maternal diabetes and ROP.^14^^,^^15^ Finally, a recent study comparing associations of GDM and hypertensive disorders of pregnancy with both the risk of developing ROP and the stage of ROP found that GDM was more strongly associated with a higher risk and greater severity of ROP than hypertensive disorders of pregnancy. However, their study did not include a control group of infants born to healthy mothers.^16^

Our multivariate logistic regression analyses found a positive association between stage 3-5 ROP and maternal DM exposure in premature infants with BW <1500 g and GA <31 weeks. The odds were highest for both type 1 and type 2 diabetes. Interestingly, several covariates in the analysis were positively associated with maternal diabetes but negatively correlated with ROP, and viceversa. For example, ROP was associated with both lower GA and BW, while diabetes exposure was associated with higher GA and BW, suggesting that these factors are not confounding the observed positive association between maternal diabetes exposure and risk of ROP progression. Other variables, such as birth location, IVH, and year of birth also showed opposing trends suggesting that maternal diabetes independently increases the odds of progression to advanced ROP stages.

Although there was a similar prevalence of diabetes exposure between the stage 3-5 group and the stage 0 group (9%) overall, we detected lower proportions of GDM among those exposed to maternal diabetes in the stage 3-5 ROP group (52%) where mean GA was 25 weeks, compared with the stage 0 group (60%) where mean GA was 29 weeks. Since GDM screening is recommended between 24–28 weeks of gestation,^30^, ^31^, ^32^ most of the mothers of infants with ROP stage 0 would have developed detectable GDM by time of birth, while infants with very low GA may have been exposed to undiagnosed GDM, biasing the results. A prospective study including early testing for GDM could help clarify this potential association by diagnosing GDM before stage 3-5 ROP children are born.

As a level IV NICU and large academic center, infants with a diagnosis of advanced ROP are often transferred to our study institution for management and treatment. We found that infants with stage 3-5 ROP were more likely to be outborn compared with controls. This is consistent with other studies that found ROP risk for inborn infants is 2/3 of that of outborn infants^33^ or observed lower rates of severe ROP in university hospital versus non-university hospital NICUs.^34^ Although the reasons for these associations are likely multifactorial (prenatal care, access to subspecialists, institutional resources), these data suggest that infants at risk of ROP who are born at smaller or nonacademic medical centers may potentially benefit from wider screening inclusion criteria, more frequent follow-up, and an improved system for early detection, monitoring, and treatment. Independent of place of birth, our sensitivity analyses in outborn and inborn cohorts maintained a positive significant association between maternal diabetes exposure and stage 3-5 ROP.

Our study has several limitations. First, it focuses on a rare outcome of stage 3-5 ROP; however, the scale of this study is equivalent to or larger than prior studies addressing this question.^14^^,^^15^ In addition, the depth of phenotyping within our curated database allowed for easy identification of the exposure and outcome of interest, as well as diabetes subtype analyses, which had not been previously reported. Second, given the high prevalence of outborn infants with stage 3-5 ROP in this study (where complete maternal EHR data are not available), information on maternal hemoglobin A1c levels was not available for analysis. Obtaining hemoglobin A1c for future studies would likely require better EHR integration across medical systems. Third, this study was limited to infants with ROP screening and maternal diabetes data, which can introduce selection bias against infants with missing data. Finally, the database is generated from manually curated EHR, which can be subject to coding errors; however, this limitation was mitigated with additional manual chart review for the outcomes of interest and missing variables.

It is important to note that this is an observational study, which is intended to test and generate hypotheses for future work but cannot determine causality. Indeed, this work provides foundational data for future exploration of the biological mechanism linking hyperglycemia to retinopathy. Future work might investigate maternal glycemic control throughout pregnancy with variables including hemoglobin A1c to determine what aspects of glycemic control (extreme levels or variability, for example) are specifically associated with an infant’s risk of ROP progression. Finally, given that maternal diabetes increases the risk of prematurity^35^ and prematurity increases the risk of diabetes,^36^ the compounded risks of these 2 vision-threatening conditions on population health should be further explored.

In summary, our study identified a positive association suggesting that neonates screened within typical BW and GA criteria and who were exposed to maternal diabetes in utero have significantly higher odds of developing stage 3-5 ROP compared to those not exposed, assuming all other controlled variables remain constant. When looking at the specific subtypes of maternal diabetes, we found a significant association between stage 3-5 ROP and maternal T1DM and T2DM. Given the increasing number of premature infants that can survive despite low GA and BW and the dearth of ophthalmologists available to screen for ROP, future inclusion of maternal diabetes in decisions regarding ROP screening intervals or participation outside of current screening guidelines may be beneficial to prevent disease progression and preserve vision in at-risk infants.
