# Supplementary material for: Exposure to Type 1 and Type 2 Maternal Diabetes is Associated with Stage 3-5 Retinopathy of Prematurity
Source: Ophthalmol Sci. 2026 Mar 4;6(6):101143. doi: 10.1016/j.xops.2026.101143 (PMC13139983; doi:10.1016/j.xops.2026.101143)
Supplement: Supplemental Table 1 [file mmc1.pdf]

Median BW (g) by ROP Stage													
	Wilcoxon Signed-Rank Test ***p-value***												
	**ROP Stage 0**			**ROP Stage 3-5**			$< 2.2 \times 10^{-16}$	ROP Stage 1-2			**ALL**		
	**median**	***LQ***	***UQ***	**median**	***LQ***	***UQ***		**median**	***LQ***	***UQ***	**median**	***LQ***	***UQ***
**ALL**	1120	910	1330	650	560	780		795	667	990	1000	766	1250
	Kruskal-Wallis Test ***p-value***												
Variable	**ROP Stage 0**			**ROP Stage 3-5**				ROP Stage 1-2			**ALL**		
**Race**	**median**	***LQ***	***UQ***	**median**	***LQ***	***UQ***	$< 2.2 \times 10^{-16}$	**median**	***LQ***	***UQ***	**median**	***LQ***	***UQ***
White	1150	946	1359	660	566	790		815	680	1020	1020	780	1270
Black	1048	830	1260	633	538	723		720	630	870	930	728	1200
Other	1145	900	1305	664	585	692		825	700	978	1049	793	1253
**Sex**	**median**	***LQ***	***UQ***	**median**	***LQ***	***UQ***	$< 2.2 \times 10^{-16}$	**median**	***LQ***	***UQ***	**median**	***LQ***	***UQ***
F	1080	870	1298	630	550	700		770	640	950	959	740	12100
M	1160	945	1360	690	580	830		825	680	1019	1032	790	1290
**Birth Location**	**median**	***LQ***	***UQ***	**median**	***LQ***	***UQ***	$< 2.2 \times 10^{-16}$	**median**	***LQ***	***UQ***	**median**	***LQ***	***UQ***
Inborn	1140	918	1335	640	550	760		820	680	1010	1050	810	1280
Outborn	1093	894	1320	660	570	786		768	652	960	913	700	1189
**Maternal DM**	**median**	***LQ***	***UQ***	**median**	***LQ***	***UQ***	$< 2.2 \times 10^{-16}$	**median**	***LQ***	***UQ***	**median**	***LQ***	***UQ***
No Maternal DM	1120	900	1330	650	560	775		793	660	981	990	760	1250
Maternal DM	1200	960	1380	660	600	795		810	700	1000	1060	810	1310
**DM Type**	**median**	***LQ***	***UQ***	**median**	***LQ***	***UQ***	$< 2.2 \times 10^{-16}$	**median**	***LQ***	***UQ***	**median**	***LQ***	***UQ***
GDM	1210	941	1375	640	575	735		810	730	1050	1055	795	1310
T1DM	1190	1090	1683	795	786	819		855	730	1015	1090	855	1225
T2DM	1200	960	1360	670	581	780		709	678	840	1040	850	1330
**NEC**	**median**	***LQ***	***UQ***	**median**	***LQ***	***UQ***	$< 2.2 \times 10^{-16}$	**median**	***LQ***	***UQ***	**median**	***LQ***	***UQ***
No NEC	1130	915	1330	660	566	780		800	670	1000	1010	780	1263
NEC	1015	833	1240	630	530	737		778	653	920	850	668	1040
**IVH****	**median**	***LQ***	***UQ***	**median**	***LQ***	***UQ***	$< 2.2 \times 10^{-16}$	**median**	***LQ***	***UQ***	**median**	***LQ***	***UQ***
No IVH	1120	910	1330	635	555	752		810	690	1000	1025	790	1270
IVH Grade 1	1210	950	1369	640	550	849		810	670	1038	1070	800	1320
IVH Grade 2	1030	838	1250	670	565	753		712	630	840	839	680	1050
IVH Grade 3	1080	860	1340	643	545	780		810	680	1085	850	653	1140
IVH Grade 4	1030	810	1230	683	621	776		760	614	910	783	643	1029
**BPD**	**median**	***LQ***	***UQ***	**median**	***LQ***	***UQ***	$< 2.2 \times 10^{-16}$	**median**	***LQ***	***UQ***	**median**	***LQ***	***UQ***
No BPD	1260	1110	1420	885	773	1054		1116	953	1380	1250	1080	1410
BPD	963	790	1170	645	560	760		760	650	900	840	680	1050
