# Supplementary material for: Exposure to Type 1 and Type 2 Maternal Diabetes is Associated with Stage 3-5 Retinopathy of Prematurity
Source: Ophthalmol Sci. 2026 Mar 4;6(6):101143. doi: 10.1016/j.xops.2026.101143 (PMC13139983; doi:10.1016/j.xops.2026.101143)
Supplement: Supplemental Table 2 [file mmc2.pdf]

Median BW (g) by Maternal Diabetes Exposure																			
	Wilcoxon Signed-Rank Test ***p-value***							**Maternal diabetes subtypes**									**ALL**		
	**No Diabetes**			**Yes Diabetes**				GDM			T1DM			T2DM					
	**median**	***LQ***	***UQ***	**median**	***LQ***	***UQ***	*0.002580*	**median**	***LQ***	***UQ***	**median**	***LQ***	***UQ***	**median**	***LQ***	***UQ***	**median**	***LQ***	***UQ***
**ALL**	990	760	1250	1060	810	1310		1055	795	1310	1090	855	1226	1040	850	1330	1000	766	1250
	Kruskal-Wallis Test ***p-value***							**Maternal diabetes subtypes**									**ALL**		
**Variable**	**No Diabetes**			**Yes Diabetes**				GDM			T1DM			T2DM					
**Race**	**median**	***LQ***	***UQ***	**median**	***LQ***	***UQ***	$5.37x10^{-08}$	**median**	***LQ***	***UQ***	**median**	***LQ***	***UQ***	**median**	***LQ***	***UQ***	**median**	***LQ***	***UQ***
White	1010	780	1270	1105	868	1333		1110	832	1333	1150	885	1249	1090	923	1355	1020	780	1270
Black	930	725	1200	920	733	1220		915	744	1204	815	685	1085	945	729	1225	930	728	1200
Other	1025	766	1250	1230	970	1315		1240	1095	1299	NA	NA	NA	970	820	1350	1049	793	1253
**Sex**	**median**	***LQ***	***UQ***	**median**	***LQ***	***UQ***	$4.48x10^{-09}$	**median**	***LQ***	***UQ***	**median**	***LQ***	***UQ***	**median**	***LQ***	***UQ***	**median**	***LQ***	***UQ***
F	950	740	1203	1070	790	1273		1085	760	1271	1090	940	1226	1025	785	1280	959	740	1210
M	1030	789	1280	1058	833	1333		1050	832	1330	1075	805	1233	1080	910	1360	1032	790	1290
**Birth Location**	**median**	***LQ***	***UQ***	**median**	***LQ***	***UQ***	$< 2.2x10^{-16}$	**median**	***LQ***	***UQ***	**median**	***LQ***	***UQ***	**median**	***LQ***	***UQ***	**median**	***LQ***	***UQ***
Inborn	1040	810	1270	1120	850	1323		1120	850	1314	1090	990	1220	1095	900	1345	1050	810	1280
Outborn	912	700	1181	930	739	1237		959	735	1270	885	806	1227	930	717	1100	913	700	1190
**ROP Stage**	**median**	***LQ***	***UQ***	**median**	***LQ***	***UQ***	$< 2.2x10^{-16}$	**median**	***LQ***	***UQ***	**median**	***LQ***	***UQ***	**median**	***LQ***	***UQ***	**median**	***LQ***	***UQ***
ROP Stage 0	1120	900	1330	1200	960	1380		1210	941	1375	1190	1090	1683	1200	960	1360	1120	910	1330
ROP Stage 1	850	707	1040	890	723	1060		1000	815	1090	880	633	1065	717	680	880	850	710	1049
ROP Stage 2	747	630	883	790	670	875		744	670	910	855	825	878	700	685	755	750	640	882
ROP Stage 3	660	560	780	650	578	786		630	550	730	795	786	819	650	570	690	660	563	780
ROP Stage 4	626	511	700	810	735	884		810	735	884	NA	NA	NA	NA	NA	NA	629	521	700
ROP Stage 5	588	554	680	1395	1395	1395		NA	NA	NA	NA	NA	NA	1395	1395	1395	595	558	892
**ROP Category**	**median**	***LQ***	***UQ***	**median**	***LQ***	***UQ***	$< 2.2x10^{-16}$	**median**	***LQ***	***UQ***	**median**	***LQ***	***UQ***	**median**	***LQ***	***UQ***	**median**	***LQ***	***UQ***
0	1120	900	1330	1200	960	1380		1210	941	1375	1190	1090	1683	1200	960	1360	1120	910	1330
1, 2	793	660	981	810	700	1000		810	730	1050	855	730	1015	709	678	840	795	667	990
3, 4, 5	650	560	775	660	600	795		640	575	735	795	786	819	670	581	780	650	560	780
**NEC**	**median**	***LQ***	***UQ***	**median**	***LQ***	***UQ***	$3.19x10^{-12}$	**median**	***LQ***	***UQ***	**median**	***LQ***	***UQ***	**median**	***LQ***	***UQ***	**median**	***LQ***	***UQ***
No NEC	1010	770	1260	1080	810	1320		1060	790	1310	1090	858	1235	1075	878	1333	1010	780	1263
NEC	840	660	1040	920	836	1023		960	838	1038	890	865	1059	850	670	950	850	668	1040
**IVH**	**median**	***LQ***	***UQ***	**median**	***LQ***	***UQ***	$< 2.2x10^{-16}$	**median**	***LQ***	***UQ***	**median**	***LQ***	***UQ***	**median**	***LQ***	***UQ***	**median**	***LQ***	***UQ***
No IVH	1020	790	1260	1080	850	1310		1060	815	1303	1080	870	1200	1070	910	1350	1025	790	1270
IVH Grade 1	1070	800	1310	1220	817	1380		1257	752	1443	1220	840	1270	1105	961	1212	1070	800	1320
IVH Grade 2	835	680	1050	955	695	1090		959	755	1035	1190	1190	1190	750	648	1028	839	680	1050
IVH Grade 3	840	650	1140	900	759	1435		1070	900	1800	NA	NA	NA	665	638	691	850	653	1140
IVH Grade 4	780	643	1034	830	715	908		830	715	908	NA	NA	NA	NA	NA	NA	783	643	1029
**BPD**	**median**	***LQ***	***UQ***	**median**	***LQ***	***UQ***	$< 2.2x10^{-16}$	**median**	***LQ***	***UQ***	**median**	***LQ***	***UQ***	**median**	***LQ***	***UQ***	**median**	***LQ***	***UQ***
No BPD	1250	1080	1410	1270	1090	1443		1310	1135	1460	1223	1085	1687	1233	1070	1390	1250	1080	1410
BPD	840	680	1040	900	730	1150		875	730	1128	940	795	1171	880	695	1048	840	680	1050
