# Supplementary material for: Exposure to Type 1 and Type 2 Maternal Diabetes is Associated with Stage 3-5 Retinopathy of Prematurity
Source: Ophthalmol Sci. 2026 Mar 4;6(6):101143. doi: 10.1016/j.xops.2026.101143 (PMC13139983; doi:10.1016/j.xops.2026.101143)
Supplement: Supplemental Table 3 [file mmc3.pdf]

Median GA (wks) by ROP Stage													
	Wilcoxon Signed-Rank Test ***p-value***												
	**ROP Stage 0**			**ROP Stage 3-5**				ROP Stage 1-2			**ALL**		
	**median**	***LQ***	***UQ***	**median**	***LQ***	***UQ***	*$< 2.2x10^{-16}$*	**median**	***LQ***	***UQ***	**median**	***LQ***	***UQ***
**ALL**	28.9	27.3	30.1	25.0	24.1	26.0		26.1	24.9	27.8	28.0	26.0	29.7
	Kruskal-Wallis Test ***p-value***												
Variable	**ROP Stage 0**			**ROP Stage 3-5**				ROP Stage 1-2			**ALL**		
**Race**	**median**	***LQ***	***UQ***	**median**	***LQ***	***UQ***	*$< 2.2x10^{-16}$*	**median**	***LQ***	***UQ***	**median**	***LQ***	***UQ***
White	28.9	27.4	30.1	25.0	24.2	26.0		26.3	25.0	28.0	28.0	26.1	29.7
Black	28.4	26.9	30.0	24.9	24.1	25.3		25.7	24.6	27.0	27.6	25.7	29.6
Other	29.0	27.6	30.2	24.8	23.9	25.1		25.5	25.0	27.6	28.3	25.8	29.8
**Sex**	**median**	***LQ***	***UQ***	**median**	***LQ***	***UQ***	*$< 2.2x10^{-16}$*	**median**	***LQ***	***UQ***	**median**	***LQ***	***UQ***
F	28.9	27.1	30.1	25.0	24.0	26.0		26.0	24.9	27.7	28.0	26.1	29.7
M	28.7	27.4	30.1	25.0	24.3	26.0		26.3	25.0	28.0	27.9	25.8	29.7
**Birth Location**	**median**	***LQ***	***UQ***	**median**	***LQ***	***UQ***	*$< 2.2x10^{-16}$*	**median**	***LQ***	***UQ***	**median**	***LQ***	***UQ***
Inborn	29.0	27.4	30.3	25.1	24.3	26.0		26.4	25.0	28.1	28.3	26.6	29.9
Outborn	28.4	27.0	30.0	25.0	24.1	26.0		25.7	24.6	27.4	27.0	25.1	29.1
**Maternal DM**	**median**	***LQ***	***UQ***	**median**	***LQ***	***UQ***	*$< 2.2x10^{-16}$*	**median**	***LQ***	***UQ***	**median**	***LQ***	***UQ***
No Maternal DM	28.7	27.3	30.1	25.0	24.1	25.9		26.0	24.9	27.8	27.9	26.0	29.6
Maternal DM	29.1	27.7	30.3	25.9	24.4	26.6		26.7	25.7	27.7	28.6	26.7	29.9
**DM Type**	**median**	***LQ***	***UQ***	**median**	***LQ***	***UQ***	*$< 2.2x10^{-16}$*	**median**	***LQ***	***UQ***	**median**	***LQ***	***UQ***
GDM	29.0	27.6	30.3	25.1	24.1	26.1		27.0	25.9	27.7	28.6	26.7	30.0
T1DM	29.6	29.0	30.4	26.0	25.9	26.3		26.9	26.3	28.1	29.0	26.9	30.0
T2DM	29.1	28.0	30.1	26.3	24.9	27.0		25.7	25.4	26.1	28.1	26.9	29.7
**NEC**	**median**	***LQ***	***UQ***	**median**	***LQ***	***UQ***	*$< 2.2x10^{-16}$*	**median**	***LQ***	***UQ***	**median**	***LQ***	***UQ***
No NEC	28.9	27.3	30.1	25.0	24.1	26.0		26.1	24.9	28.0	28.0	26.1	29.7
NEC	28.1	26.7	29.6	25.0	24.4	26.0		25.6	24.8	27.3	26.6	25.0	28.6
**IVH**	**median**	***LQ***	***UQ***	**median**	***LQ***	***UQ***	*$< 2.2x10^{-16}$*	**median**	***LQ***	***UQ***	**median**	***LQ***	***UQ***
No IVH	28.9	27.4	30.1	25.0	24.4	26.1		26.4	25.1	28.1	28.1	26.4	29.9
IVH Grade 1	29.3	27.9	30.4	25.3	24.3	26.5		26.4	24.8	28.4	28.7	26.6	30.0
IVH Grade 2	27.6	26.4	29.0	24.9	24.1	25.3		25.1	24.3	26.3	26.1	24.8	27.9
IVH Grade 3	27.7	26.6	29.0	24.9	23.9	25.1		25.3	24.1	27.0	26.0	24.9	27.9
IVH Grade 4	27.4	26.0	29.6	24.6	24.0	25.6		25.0	24.0	27.1	25.6	24.1	28.0
**BPD**	**median**	***LQ***	***UQ***	**median**	***LQ***	***UQ***	*$< 2.2x10^{-16}$*	**median**	***LQ***	***UQ***	**median**	***LQ***	***UQ***
No BPD	30.0	28.9	30.7	26.9	26.3	27.4		29.1	28.0	30.1	29.9	28.7	30.6
BPD	27.6	26.4	28.8	25.0	24.1	25.9		25.7	24.7	27.1	26.7	25.1	28.1
