# Supplementary material for: Exposure to Type 1 and Type 2 Maternal Diabetes is Associated with Stage 3-5 Retinopathy of Prematurity
Source: Ophthalmol Sci. 2026 Mar 4;6(6):101143. doi: 10.1016/j.xops.2026.101143 (PMC13139983; doi:10.1016/j.xops.2026.101143)
Supplement: Supplemental Table 4 [file mmc4.pdf]

Median GA (wks) by Maternal Diabetes Exposure																			
	Wilcoxon Signed-Rank Test ***p-value***							**Maternal diabetes subtypes**									**ALL**		
	**No Diabetes**			**Yes Diabetes**				GDM			T1DM			T2DM					
	**median**	***LQ***	***UQ***	**median**	***LQ***	***UQ***	*0.000432*	**median**	***LQ***	***UQ***	**median**	***LQ***	***UQ***	**median**	***LQ***	***UQ***	**median**	***LQ***	***UQ***
**ALL**	27.9	26.0	29.6	28.6	26.7	29.9		28.6	26.7	30.0	29.0	26.9	30.0	28.1	26.9	29.7	28.0	26.0	29.7
	Kruskal-Wallis Test ***p-value***							**Maternal diabetes subtypes**									**ALL**		
**Variable**	**No Diabetes**			**Yes Diabetes**				GDM			T1DM			T2DM					
**Race**	**median**	***LQ***	***UQ***	**median**	***LQ***	***UQ***	9.60×10^{-05}	**median**	***LQ***	***UQ***	**median**	***LQ***	***UQ***	**median**	***LQ***	***UQ***	**median**	***LQ***	***UQ***
White	28.0	26.0	29.7	28.7	26.9	30.0		28.7	27.0	30.0	29.3	27.6	30.2	28.0	26.9	29.7	28.0	26.1	29.7
Black	27.6	25.7	29.6	27.6	26.1	29.7		27.4	26.0	29.6	27.9	26.3	29.0	28.7	26.4	29.8	27.6	25.7	29.6
Other	28.0	25.7	29.6	29.1	28.9	30.6		29.8	28.9	30.6	NA	NA	NA	29.1	28.4	29.4	28.3	25.8	29.8
**Sex**	**median**	***LQ***	***UQ***	**median**	***LQ***	***UQ***	0.003379	**median**	***LQ***	***UQ***	**median**	***LQ***	***UQ***	**median**	***LQ***	***UQ***	**median**	***LQ***	***UQ***
F	27.7	25.9	29.7	28.7	26.9	29.9		28.6	26.6	29.9	29.3	28.2	30.4	28.4	26.9	29.8	27.9	25.9	29.7
M	28.0	26.0	29.6	28.4	26.6	29.9		28.4	26.8	30.0	28.3	26.3	29.8	28.1	26.4	29.7	28.0	26.1	29.7
**Birth Location**	**median**	***LQ***	***UQ***	**median**	***LQ***	***UQ***	$< 2.2 \times 10^{-16}$	**median**	***LQ***	***UQ***	**median**	***LQ***	***UQ***	**median**	***LQ***	***UQ***	**median**	***LQ***	***UQ***
Inborn	28.3	26.6	29.9	28.9	27.4	30.1		28.8	27.2	30.3	29.4	28.4	30.3	28.6	27.3	29.7	28.3	26.6	29.9
Outborn	27.0	25.0	29.0	27.1	26.0	29.6		27.3	26.1	29.6	27.3	26.0	29.0	26.9	26.4	29.6	27.0	25.1	29.1
**ROP Stage**	**median**	***LQ***	***UQ***	**median**	***LQ***	***UQ***	$< 2.2 \times 10^{-16}$	**median**	***LQ***	***UQ***	**median**	***LQ***	***UQ***	**median**	***LQ***	***UQ***	**median**	***LQ***	***UQ***
ROP Stage 0	28.7	27.3	30.1	29.1	27.7	30.3		29.0	27.6	30.3	29.6	29.0	30.4	29.1	28.0	30.1	28.9	27.3	30.1
ROP Stage 1	26.7	25.1	28.4	26.4	25.8	27.7		27.1	26.3	27.6	27.4	26.2	28.9	25.7	25.6	26.0	26.7	25.1	28.4
ROP Stage 2	25.4	24.4	27.2	27.0	25.5	27.8		27.0	25.8	27.8	26.9	26.3	27.9	25.3	24.9	26.4	26.1	24.5	27.3
ROP Stage 3	25.0	24.1	25.9	25.6	24.2	26.4		25.1	24.1	25.3	26.0	25.9	26.3	26.1	24.4	26.9	25.2	24.2	26.0
ROP Stage 4	24.8	24.1	25.4	33.2	29.6	36.8		33.2	29.6	36.8	NA	NA	NA	NA	NA	NA	25.5	24.2	25.8
ROP Stage 5	24.3	23.9	24.8	27.7	27.7	27.7		NA	NA	NA	NA	NA	NA	27.7	27.7	27.7	24.9	23.9	25.7
**ROP Category**	**median**	***LQ***	***UQ***	**median**	***LQ***	***UQ***	$< 2.2 \times 10^{-16}$	**median**	***LQ***	***UQ***	**median**	***LQ***	***UQ***	**median**	***LQ***	***UQ***	**median**	***LQ***	***UQ***
0	28.7	27.3	30.1	29.1	27.7	30.3		29.0	27.6	30.3	29.6	29.0	30.4	29.1	28.0	30.1	28.9	27.3	30.1
1, 2	26.0	24.9	27.8	26.7	25.7	27.7		27.0	25.9	27.7	26.9	26.3	28.1	25.7	25.4	26.1	26.1	24.9	27.8
3, 4, 5	25.0	24.1	25.9	25.9	24.4	26.6		25.1	24.1	26.1	26.0	25.9	26.3	26.3	24.9	27.0	25.0	24.1	26.0
**NEC**	**median**	***LQ***	***UQ***	**median**	***LQ***	***UQ***	1.08×10^{-12}	**median**	***LQ***	***UQ***	**median**	***LQ***	***UQ***	**median**	***LQ***	***UQ***	**median**	***LQ***	***UQ***
No NEC	28.0	26.0	29.7	28.7	26.9	30.0		28.6	26.9	30.0	29.1	27.4	30.2	28.6	26.8	29.8	28.0	26.1	29.7
NEC	26.4	25.0	28.4	27.2	26.6	28.9		27.8	26.7	29.5	26.6	26.4	27.9	27.4	27.0	27.6	26.6	25.0	28.6
**IVH**	**median**	***LQ***	***UQ***	**median**	***LQ***	***UQ***	$< 2.2 \times 10^{-16}$	**median**	***LQ***	***UQ***	**median**	***LQ***	***UQ***	**median**	***LQ***	***UQ***	**median**	***LQ***	***UQ***
No IVH	28.1	26.4	29.9	28.6	27.0	30.0		28.7	27.0	30.0	28.7	27.3	29.9	28.4	26.9	29.8	28.1	26.4	29.9
IVH Grade 1	28.7	26.6	30.0	29.1	26.7	30.1		29.1	26.1	30.1	29.3	26.3	30.1	29.1	27.7	29.9	28.7	26.6	30.0
IVH Grade 2	26.0	24.6	27.6	26.9	26.0	27.6		27.1	26.3	27.6	29.4	29.4	29.4	26.5	25.8	26.9	26.1	24.8	27.9
IVH Grade 3	25.7	24.9	27.9	27.4	26.4	28.1		27.4	27.4	28.7	NA	NA	NA	24.3	23.5	25.1	26.0	24.9	27.9
IVH Grade 4	25.6	24.1	27.9	25.8	25.1	27.5		25.8	25.1	27.5	NA	NA	NA	NA	NA	NA	25.6	24.1	28.0
**BPD**	**median**	***LQ***	***UQ***	**median**	***LQ***	***UQ***	$< 2.2 \times 10^{-16}$	**median**	***LQ***	***UQ***	**median**	***LQ***	***UQ***	**median**	***LQ***	***UQ***	**median**	***LQ***	***UQ***
No BPD	29.9	28.7	30.6	29.9	29.0	30.6		30.1	29.1	30.7	29.7	29.1	30.4	29.3	28.0	30.5	29.9	28.7	30.6
BPD	26.6	25.0	28.1	27.1	26.0	28.7		27.1	26.0	28.7	27.9	26.3	29.5	27.0	25.7	28.8	26.7	25.1	28.1
