# Supplementary material for: Exposure to Type 1 and Type 2 Maternal Diabetes is Associated with Stage 3-5 Retinopathy of Prematurity
Source: Ophthalmol Sci. 2026 Mar 4;6(6):101143. doi: 10.1016/j.xops.2026.101143 (PMC13139983; doi:10.1016/j.xops.2026.101143)
Supplement: Supplemental Table 5 [file mmc5.pdf]

Sensitivity Analysis	OR	L 95	U 95	P
Outborn Only	2.446	1.086	5.477	0.029569
Inborn Only	3.667	1.431	8.919	0.005
YOB > 2012	4.332	2.020	9.229	0.000146
